# Systematic Review: Land Cover, Meteorological, and Socioeconomic Determinants of *Aedes* Mosquito Habitat for Risk Mapping

**DOI:** 10.3390/ijerph14101230

**Published:** 2017-10-16

**Authors:** Mohamed F. Sallam, Chelsea Fizer, Andrew N. Pilant, Pai-Yei Whung

**Affiliations:** 1 Resilient Environment and Health, Agriculture and Water Solutions, National Exposure Research laboratory/System Exposure Division, Oak Ridge Institute for Science and Education, 109 T.W. Alexander Dr., Research Triangle Park, NC 27711, USA; 2 Oak Ridge Associated Universities, Contractor to US EPA, Office of Research and Development, National Exposure Research Laboratory, Environmental Protection Agency, 109 T.W. Alexander Drive, Research Triangle Park, NC 27711, USA; Fizer.Chelsea@epa.gov; 3 Office of Research and Development, National Exposure Research Laboratory, Environmental Protection Agency, 109 T.W, Oak Ridge Associated Universities, Alexander Drive, Research Triangle Park, Oak Ridge, NC 27711, USA; Pilant.Drew@epa.gov (A.N.P.); Whung.Pai-Yei@epa.gov (P.-Y.W.)

**Keywords:** *Aedes*, dengue, ecological modeling, physical systems, Zika

## Abstract

Asian tiger and yellow fever mosquitoes (*Aedes albopictus* and *Ae. aegypti*) are global nuisances and are competent vectors for viruses such as Chikungunya (CHIKV), Dengue (DV), and Zika (ZIKV). This review aims to analyze available spatiotemporal distribution models of *Aedes* mosquitoes and their influential factors. A combination of five sets of 3–5 keywords were used to retrieve all relevant published models. Five electronic search databases were used: PubMed, MEDLINE, EMBASE, Scopus, and Google Scholar through 17 May 2017. We generated a hierarchical decision tree for article selection. We identified 21 relevant published studies that highlight different combinations of methodologies, models and influential factors. Only a few studies adopted a comprehensive approach highlighting the interaction between environmental, socioeconomic, meteorological and topographic systems. The selected articles showed inconsistent findings in terms of number and type of influential factors affecting the distribution of *Aedes* vectors, which is most likely attributed to: (i) limited availability of high-resolution data for physical variables, (ii) variation in sampling methods; *Aedes* feeding and oviposition behavior; (iii) data collinearity and statistical distribution of observed data. This review highlights the need and sets the stage for a rigorous multi-system modeling approach to improve our knowledge about *Aedes* presence/abundance within their flight range in response to the interaction between environmental, socioeconomic, and meteorological systems.

## 1. Introduction

Mosquitoes are known vectors for disease transmission. They are globally significant with links to a million deaths annually [1,2]. Asian tiger and yellow fever mosquitoes (*Aedes albopictus* and *Ae. aegypti*) are nuisances on nearly every continent and are competent vectors in the Western Hemisphere for a number of different viruses including Chikungunya (CHIKV), Dengue (DV), and Zika (ZIKV) [3]. Both mosquito vectors are day-time biters with outdoor opportunistic feeding preference and resting behavior [4,5,6]. Their females lay drought-resistant eggs in water-holding containers (natural and man-made), estuaries, tree holes, and soil. Their eggs are usually laid either near water surfaces or on dry surfaces that may be inundated with water as a signal for hatching. In native habitats of Southeast Asia, the juveniles develop in tree holes and emerging females feed on available vertebrate hosts. The heterogeneity of numerous small habitats used by container-inhabiting *Aedes* vectors and the limited dispersal distances make their surveillance and control difficult [7,8,9]. However, international trade and fast travel has potentially expanded the dispersal range of both species to residential and urban settings throughout the Americas and Europe in the 1980s and Africa in the 1990s [10,11]. With the increased human-made developments and global urban expansion, both species have expanded their geographic range and have become increasingly associated with urban landscapes [9]. Both species have also become increasingly capable of exploiting human-made container habitats and human blood meal hosts. The spatial and temporal distribution extent of both *Ae. albopictus* and *Ae. aegypti* could predict the areas under risk of *Aedes*-related disease, especially during outbreaks. Although *Ae. albopictus* has been shown to feed on a range of vertebrate species beyond humans, the vector competency of this mosquito vector is still questionable in transmitting viral diseases to humans in areas where *Ae. aegypti* is either absent or uncommon [12,13]. Recent research has shown that transmission of *Aedes-*related diseases occurs within defined spatial and temporal patterns that depend on the mosquito’s geographic distribution range and vector capacity [14]. Distribution limits and vector capacities are greatly influenced by the biological and ecological requirements of the mosquito vectors. Accordingly, their requirements are related to an integrated complex system constituted of climate, land use-land cover (LULC), topography, and socioeconomic variables. Understanding the spatial and temporal distributions of mosquito communities and population dynamics may help in predicting transmission cycles and eventually implementing targeted surveillance and control measures [15,16]. This will help mosquito control districts in prioritizing their surveillance and control activities to efficiently target hot spot areas during outbreaks.

The increase in the US human population and urbanization, in combination with an expanding range of *Aedes* vectors, has public health departments concerned about future transmission frequencies and intensities of mosquito-borne diseases like DV, CHIKV, and ZIKV [17,18,19]. These concerns have resulted in development of distribution models for both *Aedes* vectors and their associated disease transmission frequencies and intensities. Risk maps generated from these models will eventually help in understanding the geographic distribution range of unsampled areas in order to be targeted in surveillance and control efforts. These models include biological and vectorial capacity parameters that predict competency of *Aedes* vectors. Other external variables such as environmental, topographic, and socioeconomic variables, were included to help delineate the distribution range. Few of the previous models emphasized the potentiality of climate variables, in terms of temperature and precipitation, in predicting the global and regional distribution of both vectors [18,20,21]. Although temperature demonstrates a crucial factor limiting the geographic extent of both *Aedes* vectors [18,22], these results alone are not sufficient to explain where and when these species can and cannot persist. Other models went further by using a broader range of climatic variables including precipitation, predicting the distributions of both species using statistical tools [10,23,24,25,26]. *Aedes*-borne disease mitigation efforts can be improved through reviewing these models to better encompass recent advances, delineate the potential explanatory variables that predict both species, and identify existing knowledge gaps.

The US Environmental Protection Agency, in partnership with the City of Brownsville, TX is working to identify and map hotspots of *Aedes* vectors. Transmission potential of ZIKV, DENV, and CHIKV in Brownsville exists partly due to the abundant distribution of both *Ae. aegypti* and *Ae. albopictus* vectors. The microhabitats and limited flight range of *Aedes* vectors necessitate development of a fine-scale spatiotemporal distribution map in order to better target surveillance and mitigation programs. In this paper we conducted a comprehensive literature review of published peer-reviewed methodologies, models and findings related to mitigation of *Aedes-*related disease transmission and environmental determinants for *Aedes* habitats used for developing maps of presence risk. The paper describes and summarizes the published peer-reviewed studies on *Aedes* vector ecological modeling and key habitat determinants as an input to our City of Brownsville research project design.

## 2. Methods

### 2.1. Search Query

A combination of five sets of 3–5 keywords were used to retrieve all published data on modeling of mosquito vector of ZIKV. The five keyword combinations were: (i) Land use-land cover (LULC), mosquito, modeling, socioeconomic, *Aedes*; (ii) Zika, modeling, mosquito, landscape, socioeconomic; (iii) Zika, modeling, mosquito, landscape; (iv) LULC, mosquito, socioeconomic; and (v) niche modeling, mosquito, USA. Five electronic databases were used to search for key words: PubMed, MEDLINE, EMBASE, Scopus, and Google Scholar through 17 May 2017. Only English articles were considered in the search query. For the MEDLINE database, the MeSH browser tool was used to narrow the search query and retrieve relevant published articles.

### 2.2. Hierarchical Decision Tree for Article Selection

**Figure 1:**
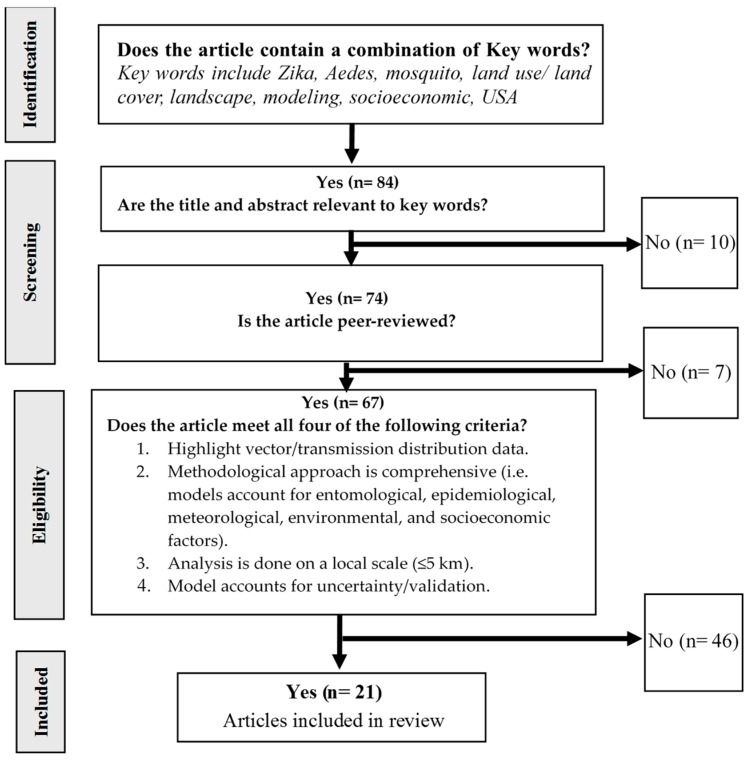
Hierarchical decision tree used in article selection.

A hierarchical decision tree was constructed to select articles for review (Figure 1). First, all published articles highlighting *Aedes*, mosquitoes, land use/land cover, socioeconomic data, climate, risk maps, spatial analysis, modeling, and prediction (*n* = 84) were considered for preliminary screening. All article titles and abstracts were reviewed for relevance to the key words. Articles addressing any ecological or epidemiological characteristics of Zika outbreaks, including studies regarding previous outbreaks and regions outside the Americas, were considered as relevant (*n* = 74).

The relevant articles were categorized into either peer-reviewed (*n* = 67) or not (*n* = 7). Peer-reviewed articles were considered for further analysis if they covered the following selection criteria: (1) highlights vector/seroprevalence distribution data and their response to the interaction between different systems (natural, physical, and socioeconomic); (2) comprehensive, in terms of utilizing a multidisciplinary approach incorporating entomological, epidemiological, climate, and socioeconomic models; (3) local scale using highly spatially resolved data layers; (4) model calibration/uncertainty and threshold indicators (e.g., AICc, *P,* R^2^) used to validate these models. Peer-reviewed and relevant articles not covering all four selection criteria were excluded (*n* = 46). Key articles were identified as articles focusing on spatial and temporal distribution ranges/limitations of ZIKV and/or mosquito vectors in response to their predicting natural, physical, and socioeconomic systems.

Articles were excluded if their titles and abstracts did not match our search query mentioned above. Review articles, non-peer-reviewed articles (theses and dissertations), editorials, letters to the editor, recommendations and guidelines, duplicate studies, and internal reports were excluded unless they contained an original and relevant analysis. Additionally, laboratory and in vivo models were excluded.

## 3. Results and Discussion

### 3.1. Relevant and Key Articles

Out of 84, a total of 74 articles were relevant to our literature search. These articles highlighted mosquito-related diseases and included key words as part of the publication title and abstract. Seven of the relevant articles were either theses, dissertations, review articles, or governmental reports, and were therefore excluded. Of the 67 relevant peer-reviewed articles, only 21 met the four selection criteria and were considered as “key articles” for our systematic review (Table 1). We identified and summarized the explanatory variables and data types used in the 21 key articles: entomological/incidence, meteorological, socioeconomic, environmental, and topographic variables (Table 2). Nine and eleven studies were conducted in [27,28,29,30,31,32,33,34,35,36,37] and outside the US [38,39,40,41,42,43,44,45,46], respectively, highlighting spatial and/or temporal distribution patterns of entomological and epidemiological indicators. A single article highlighted the generic analysis of force of infection of mosquito-borne diseases encompassing human immunity using mathematical simulation modeling [47].

The spatial and temporal variation in mosquito vector biology and ecology, and the socioeconomic and meteorological characteristics of their preferred habitats underpin the adopted approaches of selected articles. Type, scale (local, regional, and continental), resolution of available data layers and adopted analysis methods used in the key articles may largely influence the variation in article findings (Table 2).

### 3.2. Dependent Variables

#### Entomological/Incidence Data

Variation in sampling methods of entomological and epidemiological data determined the research design adopted in selected articles. Presence and abundance of mosquito vectors were used in 14 key articles to model their distribution in response to predicting factors [27,28,29,30,31,32,33,34,37,39,41,42,44,48]. These studies utilized data of one or more entomological development stages, which were collected using different sampling techniques. Eleven out of 14 temporal analysis studies also utilized chronologically-collected datasets to represent sufficient replicates on the time-scale. Disease incidence and infection rates were used in eight of the key articles to highlight hot spot areas at which transmission potential exists [31,35,38,40,45,46,47]. However, the disease incidence and human case data do not reflect vector-host contact (VHC) ratios and the likelihood of infection rate.

Different sampling methods or chronologically-sampled data were usually used either to highlight the development rate of mosquito vectors or to maximize spatiotemporal sampling replicates (Table 2). However, the differences in mosquito presence and abundance records collected by different sampling methods and seasonal fluctuations in mosquito population flux usually generate statistical heterogeneity in entomological datasets [29,32,49]. This may cause unreliable correlations between response and explanatory variables and result in inaccurate distribution maps of mosquito presence and abundance. Landau and van Leeuwen [29] and Rey et al. [32] used log-transformed entomological data to maintain homogeneity over time between different sampling methods. Monaghan et al. [37] utilized different lifecycle stages of mosquito vectors, their approach addressed the development rate from egg to adult using simulation models for all stages as a function of daily meteorological inputs. Robert et al. [34] adopted a different approach in their model. Usually abundance of mosquito vectors and disease incidence data do not reflect actual risk to human populations, unless this abundance is coupled with biting rates or VHC [50]. Therefore, Robert et al. [34] utilized VHC ratios as a precursor for biting and infection rates, which eventually could help in determining the actual transmission risk.

Additionally, to assess the suitable microhabitats of *Aedes* vectors in response to their explanatory variables, sampling site buffer radii are needed. These buffer radii serve two purposes: (i) explain the active flight range of *Aedes* vectors; and (ii) help in building reliable correlations between extracted values of explanatory variables and *Aedes* density within these buffer radii. Although eight of the key articles extracted entomological data within a 2 km flight range of *Ae. aegypti* and *Ae. Albopictus* [29,30,31,32,33,35,39], their flight range was recorded to be a few meters rather than kilometers (~100 m) [51,52].

### 3.3. Explanatory Variables

#### 3.3.1. Meteorological Data

A growing number of articles suggest that population density of both *Ae. aegypti* and *Ae. albopictus* exhibit interannual fluctuations that are directly linked to climatic variability [53,54,55,56]. The meteorological variables were highlighted in a total of ten of the selected key articles. Generally, these studies suggest the importance of both rainfall and vegetation at regional and local levels in predicting *Ae. aegypti* and *Ae. albopictus* presence at a household scale [57,58]. Rainfall demonstrated a significant importance in increased numbers of outdoor rain-filled containers such as tires and backyard junk which may provide oviposition sites for *Ae. aegypti,* along with mean annual land surface temperature during daytime [38]. Additionally, the average minimum night-time temperature was reported to be the only significant meteorological factor that predicts increased risk of dengue fever (DF) infection with a decreasing minimum night-time temperature [43]. The DF incidence increased by 64% in areas with night time temperature below 10 °C, compared to areas with higher temperatures [43]. This was triggered by the indoor biting behavior of *Aedes* mosquito and linked to schools and work areas. Rainfall also helps in promoting vegetation as a sugar source and as a resting place for adult mosquito vectors upon emergence and post-blood feeding.

Drought-like conditions may have an indirect influence on *Ae. aegypti* populations by increasing the number of household artificial containers for water storage and serve as larval breeding sites [59,60]. Relative humidity was shown to affect body fluid loss of *Ae. albopictus*, which may explain the importance of average relative humidity in modeling daily presence and absence of this mosquito vector [61]. Five key articles addressed the drought index, relative humidity, potential evapotranspiration and soil moisture in limiting the distribution of *Ae. aegypti* and *Ae. albopictus* (Table 2). Eggs of these two mosquito vectors can survive for a few years in a drought while waiting for suitable conditions before hatching [6]. However, drought significantly affects availability of water resources for adult mosquito oviposition, development and survival rates of immature stages and adults, respectively [6]. Drought negatively affects the outdoor water availability at the time it may trigger the increase of indoor water containers. Seasonal drought may give us information about time needed to prepare for targeted control measures at areas under risk of these mosquito vectors.

#### 3.3.2. Socioeconomic Data

The major socioeconomic data variables utilized in the selected publications were population density, household age, income level, education level, housing density, housing age, and housing structure type [30,40]. Population density, house age, income level, proportion of vacant houses, housing density and house structure variables were shown to play a significant role in predicting spatial distribution of *Ae. aegypti* presence and abundance [9,35,38,44,62,63,64]. Education level, residence time in neighborhood, and community effort in environmental management were negatively correlated with mosquito vector presence and abundance [30,40]. In contrast, Wijayanti et al. [43] demonstrated that highly-educated individuals and people employed as village-level civil servants were significantly more at risk of DF transmission.

Zhou et al. [44] demonstrated that mosquito abundance was significantly higher in older houses and in deforested areas. On the contrary, Lockaby et al. [30] reported that housing age was negatively associated with risk of vector-human contact ratios. Household age was shown to be negatively associated with abundance of *Ae. aegypti* and frequency of DF incidence [65]. However, the type of housing that is linked to housing age was not highlighted in these studies and my caused this discrepancy in previous studies. Residential and commercial areas were found to be positively associated with *Ae. aegypti* abundance in a mixed residential agricultural community [31] and an urban ecosystem [65]. In Koyadun et al. [40] house window screens are highlighted as a potential indicator for increased vector-host contact ratios and biting rates. This finding was confirmed in other studies and showed a positive association with increased DF incidence, reflecting the exophagic preference for *Ae. aegypti* in the highlighted study areas [40,66]. However, the feeding preference of the same mosquito vector may show some spatial and/or temporal resilience depending on the available ecological and food resources. Accordingly, Thammapalo et al. [67] demonstrated that houses with window screens provided preventive benefits and showed reduction of risk in association with vector-human contact.

Some variables that may help in understanding disease transmission potential are imported cases and human host immunity. The ratio of susceptible human populations to recovered ones and the ratio of infected imported human cases to the total population size may explain the transmission intensity of mosquito-borne diseases. The lack of knowledge about human immunity triggered development of some helpful mathematical simulation models [45,47] to estimate force of infection of ZIKV in travelers to and from epidemic areas. However, no actual observed data were available to validate the outcomes of these models.

#### 3.3.3. Environmental Data

Eight articles utilized 1 m pixel resolution environmental predictors such as vegetation, degree of urbanization, mixed residential areas, ecotype, normalized difference vegetation index and enhanced vegetation index. These predictors were used as indicators for the likelihood of mosquito-suitable habitats and disease incidence and infection rates.

Abundance of *Ae. aegypti* was positively correlated with urbanization-related variables such as buildings and negatively associated with habitats reflecting rural or bare land, canopy and mixed vegetation, and unpaved road [32]. Density of *Ae. albopictus* was correlated with open rain-filled artificial containers in residential areas compared with forested and wooded areas [68,69]. However, almost 27% of positive collection sites for *Ae. aegypti* were not associated with houses in other studies [28,39,46]. Other studies characterized the influence of degree of urbanization on the likelihood of repeated DF transmission [38,40,43]. For example, it was recorded in Thailand and Indonesia that DF frequency was associated with combined residential, commercial, and densely-populated urban-residential areas [40,43]. As illustrated above, density of *Ae. aegypti* and *Ae. albopictus*, and their associated diseases showed variation in their response to urbanization and vegetation variables. This variation may be modified by the human socioeconomic and behavioral contexts [43].

Additionally, topographic variables were utilized in some studies to demonstrate their potential in predicting the elevation limits of the distribution of *Culex* and malaria vectors [31,38,44,48,70]. Other elevation-related indicators such as hill-shade, aspect, curvature, and slope have been used as predictors for water catchments and possible *Culex* breeding sites [36,38].

## 4. Modeling Approaches

Some of these models attempted to maximize the sampling effort through extracting microhabitat characteristics within the active flight ranges of highlighted mosquito vectors [29,30,31,32,33,35,36,39]. In these articles, different modeling approaches were adopted to delineate spatial and temporal dependency of entomological and/or epidemiological parameters on their influential factors. Generally, these approaches utilized mechanistic and correlative models encompassing statistical regression analyses, species distribution models, and mathematical simulation models.

Regression analysis, either linear or logistic, was used to identify the most influential factors in predicting spatial and temporal distribution of vector presence and abundance [28,29,30,31,32,33,36,39,41] or disease incidence and infection rates [35,40,44]. Five of these articles used stepwise multiple regression analysis to reduce collinearity between explanatory variables and to avoid model over-fitting, especially in local studies within the county level (Table 2). In this approach, explanatory variables are entered in the regression analysis stepwise, either forward or backward, estimating the model gain (R^2^), regression coefficient (β), and corrected Akaike’s Information Criterion (AICc) with and without the newly entered variables. According to these indicators, models with the lowest AICc and highest R^2^ values were used to select the significant influential factors and to remove “noise” variables. The stepwise elimination process consists of iteratively removing these noise variables and observing the effect on the error rate of the model. Although the stepwise statistical approach is more sound than the enter method, the analysis depends on data normality and a linear correlation between response and explanatory variables [71]. Landau and van Leeuwen [29] and Rey et al. [32] log transformed the response variables (entomological/prevalence), and used the arcsine square root of explanatory factors to maintain statistical normality for data variables.

A species distribution model (SDM) approach was adopted in three articles to predict relative spatial and temporal distribution of *Ae. aegypti* and *Ae. albopictus* using limited presence-absence records of these mosquito vectors. Although there are plenty of SDM tools, Maximum Entropy (MaxEnt) and Boosted Regression Tree (BRT) are the most widely used packages [72,73,74,75]. The MaxEnt has been shown to efficiently predict species distribution even with small numbers of presence records, as compared to other SDM tools [72,74,75]. These tools depend on using presence records of species to build a correlation matrix between these records and their associated explanatory variables. The generated correlation matrix is eventually used to produce a habitat suitability map for this species. These suitable habitats in the generated maps are similar to those previously sampled locations. Many indicators are used to validate the prediction gain of SD models such as area under the curve, sensitivity vs. specificity, omission rate at 10% training presence, Kappa and TSS values [75]. However, due to limited access to many study sites, especially during outbreaks, few studies used independent field validation points to evaluate the generated risk maps [37,76]. Monaghan et al. [37] out of 21 selected articles, used independently-collected entomological datasets to validate their spatiotemporal distribution model highlighting life stage development using Skeeter Buster and DyMSiM models.

Human immunity to mosquito-borne diseases, and human movements have not been extensively highlighted in previous models due to data limitations. This limitation resulted in generating simulation models utilizing disease incidence data from previous outbreaks [45,47]. Some of these models used differential equations to estimate the force of infection as a function of recovered or susceptible human-hosts, as in Manrique et al. [47]. Massad et al. [45] estimated the force of infection as a function of the incidence rate of the total population size during an outbreak, regardless of human immunity. Although these studies are helpful in evaluating theoretical transmission potential of *Aedes-*related diseases, their findings are not validated and cannot be applied elsewhere, due to heterogeneity of human susceptibility and variation of vector-host contact ratios.

## 5. Conclusions

The spatiotemporal distribution of *Aedes* vectors is determined by interaction between environmental, socioeconomic, and meteorological variables. The influence of these variables on *Ae. aegypti* and *Ae. albopictus* presence and abundance varied amongst studies. Meteorological variables that were used to explain the distribution range of these mosquito vectors and their associated diseases include annual precipitation, mean annual day time temperature, night time temperature, drought index, relative humidity, evapotranspiration, and soil moisture. Studies show inconsistent findings about environmental variables, particularly about the influence of urban and mixed residential settings versus bare land, canopy and mixed vegetation on *Aedes* vectors. Similarly, studies have inconsistent results about socioeconomic variables. Particularly, house age (old versus new), house window screens (with and without), and household age (below 18 years and over 18) showed spatial variation in their influence on *Aedes* vectors. Contradictory findings about influential variables are most likely attributed to the variation in either sampling methods and/or feeding and oviposition behavior of *Aedes* vectors in the context of continuous interaction between geographic locations, meteorological, environmental, human behavior, and socioeconomic systems. The limited availability of high-resolution physical variable data in previous investigations may also explain this discrepancy in capturing the significant influential factors that predicts *Aedes* distribution within their flight range. Reviewed articles applied different flight ranges, often at kilometer scale, based on species entomology, biology and life cycles. However, *Aedes* vector flight range studies show they generally fly an average of several hundred meters, rather than kilometers.

The discussed investigations highlight the significance of having comparable numbers of both explanatory variables and entomological/disease incidence sampling points in order to have a reliable model. Having incomparable numbers generates “noise” due to redundancy of explanatory variables and increases error in the prediction model. Incomparable numbers of variables may also result in data collinearity, which affects number and percent contribution of the influential factors and reliability of the model. To eliminate redundant variables and reduce collinearity, regression analysis was used as a statistical tool. Additionally, data normality of both response and explanatory variables are critical to have statistically homogenous data as a preparatory phase for regression analysis. Data transformation was widely used in several studies to maintain normality, improve reliability of regression models, and minimize prediction errors.

Species distribution models were widely used to predict either relative or absolute spatio-temporal distribution of invasive and endangered species. The SDM toolbox has plenty of tools such as MaxEnt, Artificial Neural Network, Genetic Algorithm for Rule set Production, BRT, Random Forest, and Support Vector Machines. However, MaxEnt and BRT are the most widely used tools in predicting distribution range of mosquito vectors and their associated diseases. MaxEnt was shown to efficiently predict species distribution even with small numbers of presence records, as compared to other SDM tools. Many threshold indicators in MaxEnt also give the ability to validate the prediction gain of generated risk maps such as area under curve value, Jackknife test, sensitivity vs. specificity, omission rate at 10% training presence, Kappa and True Skill Statistic scores.

All but six reviewed models either focused on selected influential variables, rather than a comprehensive systems approach, or utilized sampling points, rather than sampling habitats within the *Aedes* flight range. The findings and conclusions presented in these models are therefore grounded in a biased hypothesis. One of the most important findings from our review is the variation in effects of influential factors on *Ae. aegypti* and *Ae. albopictus* behavior and ecology and eventually their spatial and temporal distributions. This variation in spatio-temporal distribution of *Ae. aegypti* and *Ae. albopictus* make it unlikely that a single system could describe the relationship between these mosquito vectors and their predicting variables.

**Figure 2:**
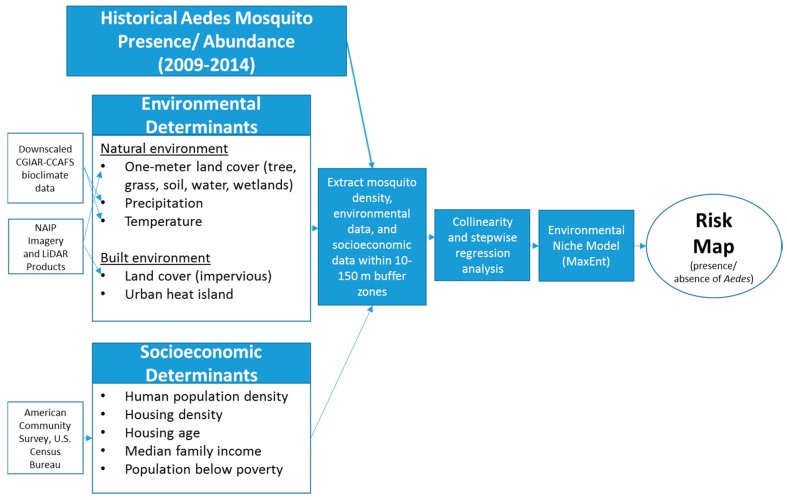
Research design for generating a risk map of *Ae. aegypti* presence/abundance in city of Brownsville, TX. CGIAR-CCAFS: Consultative Group for International Agricultural Research-Climate Change Agriculture and Food Security; NAIP: National Agriculture Imagery Program.

In our future Brownsville, TX project research design, we will emphasize the importance of a multi-systems approach to highlight the interaction between different determinants and presence/abundance of adult *Aedes* vectors within the flight range of *Aedes* vectors. Due to limitations of diseases incidence data in the city of Brownsville, human cases data were not included in our research approach. We will use meter-scale urban land cover data in integration with socioeconomic and meteorological variables (Figure 2) [77]. Seven proposed buffer zones will be generated and defined by radii (10–150 m at 20 m intervals) representing different possible flight ranges of *Aedes* vectors. Data of explanatory variables will be extracted within these generated buffer zones. Both *Aedes* data records and explanatory data variables will be log10 transformed to maintain statistical normality and minimize prediction errors. Moreover, to reduce redundancy and collinearity of utilized explanatory variables, regression analysis will be used to evaluate the influential factors that predict *Ae. aegypti* and *Ae. albopictus* abundance. Subsequently, these influential factors will be used to predict distribution of both vectors’ presence using Maximum Entropy (MaxEnt) software to map *Aedes* presence hot spots in our research study area. Mapped hot spots can be incorporated into integrated vector management strategies to reduce *Aedes* vector transmtted disease risk.

## Figures and Tables

**Table 1:** List of publications selected for the systematic review.

Reference	Model Type	Threshold/Validation Indicators
Buckner et al. [27]	Mechanistic, Priori RA	β, P, AICc-values
Hayden et al. [28]	MLM	AUC, ROC, QIC-values
Landau and van Leeuwen [29]	SMRA	R^2^, CV, residual plot,
Lockaby et al. [30]	SMRA	β, R^2^, *p*-values
Reiter and LaPointe [31]	MLM	AICc values
Rey et al. [32]	SMRA, PCA	β, Z, *p*-values
Richards et al. [33]	RA, Kriging	R^2^, CV, residual plot, predicted vs. observed, goodness-of fit, RMSSE
Robert et al. [34]	GLS	Pr, *p*-values
Rochlin et al. [35]	MLM	β, *p*-values
Sallam et al. [36]	SMRA, MaxEnt	AICc, AUC, ROC, CV, β, *p*-values
Monaghan et al. [37]	SB, DMSiM	Mean of ensemble models, mean of two life stages model
Ashby et al. [38]	BRT	RMSE, Pr, *p*-values
Gleiser and Zalazar [39]	RA	R^2^, *p*-values
Koyadun et al. [40]	ANOVA, MLM, LR, WT	R^2^, *p*-values
Rubio et al. [41]	GLMM, ML	R^2^, *p*-values
Troyo et al. [42]	ANOVA	P, Kappa values
Wijayanti et al. [43]	BPSA, INLA	IRR, DIC, predicted vs. observed
Zhou et al. [44]	SA, GI, SMRA	*p*-values
Massad et al. [45]	MDM	NA
Messina et al. [46]	BRT	AUC value, 10% omission rate value, CV
Manrique et al. [47]	SIR, SIS	NA

AICc: Akaike’s information criterion; ANOVA: analysis of variance; BPSA: Bayesian Poisson spatial analysis; BRT: boosted regression tree; CV: cross-validation; DIC: deviance information criterion; DMSiM: DyMSiM model for mosquito life stage; GI: Getis Index; GLMM: generalized linear mixed model; GLS: generalized least squares; INLA: integrated nested Laplace approximate; IRR: incidence risk ratio; LR: likelihood-ratio; MaxEnt: Maximum Entropy; MDM: mathematical differential model; ML: Maximum likelihood; MLM: multivariate logistic model; PCA: principal component analysis; Pr: Pearson correlation coefficient; QIC: quasi-likelihood under the independence model criterion; RA: regression analysis; RMSE: root mean square error; RMSSE: root mean square standardized error; SA: spatial autocorrelation; SIR: susceptible infected recovered; SIS: susceptible infected susceptible; SMRA: stepwise multiple regression analysis; SB: skeeter buster model for mosquito life stages; WT: Wald’s test.

**Table 2:** Entomological/Incidence, meteorological, socioeconomic, environmental, and topographic data variables addressed in the selected key articles.

Reference	Entomol./Inc. *	Meteorology *	Socioeconomic **	Environment **	Topography **
Buckner et al. [27]	A	P, T, RH, DI	NA	10 (1 m, aerial)	NA
Hayden et al. [28]	E	T, RH	6	2 (1 m, Ikonos-aerial)	NA
Landau and van Leeuwen [29]	log A	NA	NA	Sq. root 11 (1 m, NAIP, aerial, LiDAR)	NA
Lockaby et al. [30]	A	P, T, PET, SM	2	7 (1 m, aerial)	NA
Reiter and LaPointe [31]	A, IR	P	NA	4 (30 m, LSTM)	Elevation
Rey et al. [32]	log E, log L	NA	NA	Arcsine sq. root 17 (1 m, aerial)	NA
Richards et al. [33]	E, A	P, T	NA	2 (1 m, Ikonos)	NA
Robert et al. [34]	VHR	NA	2	NA	NA
Rochlin et al. [35]	DIn3	NA	4	7 (30 m, USGS, MODIS)	NA
Sallam et al. [36]	A, Ser.	P, T	1	2 (250 m, MODIS, USGS)	5
Monaghan et al. [37]	E, L, P, A	P, T, RH	2	NA	NA
Ashby et al. [38]	DIn1	LST, nLST	1	9 (250 m, MODIS)	Elevation
Gleiser and Zalazar [39]	A	NA	NA	4 (30 m, LSTM)	NA
Koyadun et al. [40]	DIn1	NA	22	4 (household level)	NA
Rubio et al. [41]	L	NA	NA	1 (30 m, LSTM)	NA
Troyo et al. [42]	L	NA	NA	5 (0.5–15 m, QB-ASTER)	NA
Wijayanti et al. [43]	DIn1	P, LST, nLST	53	1 (1 km, MODIS)	NA
Zhou et al. [44]	L	NA	2	4 (1 m, Ikonos)	Elevation
Massad et al. [45]	DIn2	NA	4	NA	NA
Messina et al. [46]	DIn2	P, T, RH	1	1 (5 km, MODIS)	NA
Manrique et al. [47]	DIn2	NA	1	NA	NA

* A: adult mosquitoes; DIn1: disease incidence of Dengue; DIn2: disease incidence of ZIKV; DIn3: disease incidence of WNV; DI: drought index; E: mosquito eggs; Entomol./Inc.: entomological/incidence; IR: infection rate; L: mosquito larvae; LST: land surface temp.; LSTM: Landsat TM; nLST: night land surface temp.; P: precipitation; PET: potential evapotranspiration; QB: QuickBird; RH: relative humidity; Ser: seropositive data; SM: soil moisture; T: temperature; VHR: vector-host ratio. **: the numbers refer to the number of socioeconomic, environmental, and topographic variables.
